# Culturally-tailored cookbook for promoting positive dietary change among hypertensive Filipino Americans: a pilot study

**DOI:** 10.3389/fnut.2023.1114919

**Published:** 2023-04-20

**Authors:** Madelyn O. Sijangga, David V. Pack, Nicole O. Yokota, Morgan H. Vien, Alexander D. G. Dryland, Susan L. Ivey

**Affiliations:** Health Research for Action, School of Public Health, University of California, Berkeley, Berkeley, CA, United States

**Keywords:** hypertension, dietary change, culturally-tailored intervention, lifestyle change, cardiovascular disease, culinary medicine, cardiometabolic disease, Filipino cookbook

## Abstract

**Introduction:**

Among all Asian American subgroups, Filipino-Americans have consistently been shown to have the highest rates of hypertension, raising risks of heart attack and stroke. Despite this alarming fact, little has been done to investigate culturally-sensitive interventions to control hypertension rates in this vulnerable population. To address the lack of culturally-relevant lifestyle options for blood pressure management currently available to the Filipino community, this exploratory pilot study used a design thinking approach informed by culinary medicine to develop a culturally-tailored, heart-healthy, and low sodium recipe cookbook for Filipino Americans with hypertension and evaluate its feasibility as a hypertension intervention.

**Methods:**

Our team developed a cookbook using participatory methods and design thinking, utilizing input from five Filipino culinary experts and a Registered Dietitian. The cookbook incorporates traditional Filipino recipes, excerpts from community members’ interviews, and nutrient analyses. Twenty Filipinx-identifying individuals* who self-reported physician-diagnosed hypertension were recruited from Filipino community-based organizations, enrolled into this study, provided with the cookbook, and asked to cook at least one recipe. Pre- and post-intervention surveys were conducted and centered around behavior change and features of the cookbook.

**Results:**

This study provided evidence for the cookbook’s acceptability and feasibility, with participants’ open-ended responses revealing that the recipes, nutrition labels, illustrations, and cultural aspects of the cookbook increased motivation to achieve dietary change, including reducing sodium in their diet to improve their blood pressure. Participant responses also indicated positive behavior change as a result of using the cookbook, with participants reporting increased likelihood of adopting recommended actions to lower their BP after utilizing the cookbook ($\bar{x}$ = 80.83%), compared to before ($\bar{x}$ = 63.75%, *p* < 0.008), according to Hypertension Self-Care Management scaled scores.

**Discussion:**

In conclusion, the results of this pilot study demonstrated acceptability of this unique cookbook and provide preliminary findings consistent with increased motivation in participants to make dietary changes and improve personal health, drawing attention to the importance of considering future culturally-tailored health interventions. Next steps should include a robust, randomized controlled trial design comparing measured blood pressure outcomes of an intervention vs. control group. *Filipinx is an inclusive term representing the gender identities of all participants in our study.

## 1. Introduction

Filipino Americans account for the third largest Asian American population in the U.S (1, 2). For consistency in this paper from here on, we will refer to this specific Asian subgroup as Filipino Americans. This population consistently shows disproportionately higher rates of hypertension in comparison to non-Hispanic White adults in the US (3, 4). Within the U.S. population, non-Hispanic Asian males and females have a prevalence of high blood pressure of 51.0 and 42.1%, respectively (5). However, when data are disaggregated by subpopulation, among all Asian American subgroups, Filipino Americans suffer from the highest rates of hypertension (3, 4). Studies have estimated that three in five Filipino American adults have a diagnosis of hypertension (6) and that more than half of Filipino Americans aged 50 and over will develop hypertension (7). Despite this alarming disparity, little research has been done to investigate culturally sensitive interventions to help control high blood pressure or to reduce rates of hypertension in this community. A recent study also found that hypertension awareness, treatment, and control rates among Filipino Americans were shockingly low, indicating that a majority of Filipino Americans with hypertension experience poor hypertension management (8).

Poor medical management and control of this chronic condition, as well as a variety of cultural factors, may contribute to the high rates of uncontrolled hypertension in Filipino Americans. These cultural factors include the traditional custom of sharing meals contributing to difficulties in reducing caloric intake (9) and limited medical visits due to patients not visiting until their condition is more advanced (9). Furthermore, there is a high prevalence of smoking among Filipino Americans (17%) (10), paired with a decreased likelihood of smoking cessation in a social setting due to the Filipino value of *pakikisama* (desire to get along with others to prevent conflict) (11). One major factor contributing to high prevalence of hypertension among Filipino Americans is dietary intake. The “westernized” Filipino diet consists of foods high in sodium which can exacerbate hypertension, foods high in fats, and readily digestible, refined carbohydrates, which can contribute to worsening cardiometabolic conditions (12, 13). Studies have found that the Filipino diet consists of an average of 12 g of sodium–eight times higher than recommended by the American Heart Association (10). In an attempt to help reduce and control blood pressure in hypertensive Filipino Americans, many physicians may recommend generic lifestyle modifications. Furthermore, physicians recommend dietary modifications that gravitate toward traditional American cuisine, such as the widely recommended Dietary Approaches to Stop Hypertension (DASH) diet that discourages intake of refined carbohydrates, such as white rice (14), that is reported to be a central part of the daily Filipino diet (15, 16). Existing literature has also found that patient-centered, culturally-sensitive patient-physician communication is severely lacking for ethnic minority populations (17, 18), with multiple case studies highlighting the tendency of providers to recommend the removal of cultural foods from patients’ diets completely (19). As a result, many Filipino Americans struggle with making modifications to their diet because they do not know how to, or simply do not want to modify their diet in this way. Certain dietary changes may compromise the taste of traditional Filipino food that could make adherence more difficult (16). In addition, some patients express they want to eat the same foods as family members or are concerned that making changes in diet will of necessity exclude other family members (20). These modifications are thus perceived to reduce what is a critical family joy of eating all together, a value that is especially prevalent within the Filipino American community (15). Therefore, there is a need for more culturally-tailored approaches to encourage dietary change in hypertensive Filipino Americans struggling with blood pressure management, especially support for adopting lifestyle changes.

Despite the clear necessity for more culturally nuanced dietary approaches to address high rates of hypertension in the Filipino community, few studies have been conducted to help solve this problem. While culturally-nuanced training has been provided by the Filipino American Cardiovascular Health Conference on best practices for physician interaction and counseling for Filipino patients, it has not been published (21). In our review of existing literature, we were able to find only one study in which the researchers demonstrated that a culturally-tailored educational intervention that promotes physical activity and reduces dietary sodium intake among Filipino Americans resulted in a significant reduction in blood pressure in comparison to the control (22). Other culturally-sensitive dietary interventions for Filipino Americans found were focused on non-heart related diseases, including cancer (23) and diabetes (24). Other interventions which apply a cookbook as part of their dietary intervention include ones that targeted African Americans for healthy dietary change (25), or Mexican-Americans for decreased sodium consumption and increased fruits and vegetable intake (26). Similarly, single recipes or cooking demonstrations have been part of multi-component interventions to decrease meat consumption (27) or increase purchasing of produce, including for Latino/a populations (28). The positive dietary change results from these dietary interventions, along with specific evidence from the DASH trial (29) showing diets which emphasize fruits, vegetables, and low sodium foods reduce blood pressure in hypertensive patients, serve as the rationale behind our intervention design to target a unique population and support the promise of culturally-tailored interventions.

Culinary medicine interventions include clinical and public health approaches for food-based education and skill training to increase access to disease-preventing and higher quality foods (30–32). It encompasses the aforementioned dietary change principles and may positively impact hypertension outcomes. Studies have found that culinary medicine approaches are low-cost and high-impact (33), have improved dietary attitudes, culinary skills, and dietary intake, and can increase self-efficacy and competence in nutrition knowledge (32, 34)–all of which are found to be important for blood pressure control (35, 36). While this intervention type has been shown to be successful, studies have also highlighted the need for cultural competency in this setting, with findings of higher diet adherence, improvements in disease management, and lower chronic disease burden when cultural elements are incorporated (37). Similar to other dietary intervention studies found in our review of existing literature (23, 24, 28), culturally-focused culinary medicine studies have predominantly been conducted in Hispanic/Latinx populations and in populations with diabetes (75%) (30, 37). Despite its importance, studies have highlighted a gap in culinary medicine interventions focused on Asian populations–despite recognition of this population’s increased cardiovascular disease risks (37). Despite this lack of literature, a nascent culinary medicine movement is seen to be rising in popularity within the Filipino community, with an established group of more than 2,000 members predominant on social media (38). These findings further highlight the need for the implementation and robust study of similar interventions in hypertensive, Filipino-based communities. Overall, the successes from these prior studies further support applications of design thinking to create a culturally-appropriate culinary medicine cookbook intervention for Filipino-Americans with hypertension.

In addition to the previously mentioned desire to reduce the gap in this field of heart-healthy nutrition research, the personal connection of one of our first authors (D. Pack) to this specific topic serves as inspiration for this project and the writing of this manuscript. Within the cookbook, Pack recounts a specific personal story involving his grandfather (or Lolo in Tagalog) that served as inspiration for the creation of this unique cookbook intervention; we share a summarized version of that with readers here:

“Mr. Bianes, your blood pressure is extremely high. I am going to recommend that you modify your diet and perhaps cut Filipino food out completely because it’s too unhealthy.” I remember my Lolo’s (grandfather’s) physician saying this as I sat in on one of his doctor’s appointments. I simply could not come to terms with the notion that cutting out ALL Filipino food from our diets is the solution to improving diet-related chronic health conditions like hypertension, diabetes, or cardiovascular disease. I felt determined to explore culturally sensitive solutions that allow Filipino patients to continue eating foods that are true to their roots of Filipino culture and cuisine while simultaneously improving their health. My hope is that this cookbook will provide others with the tools needed to embark on their own journey of improving health and rediscovering Filipino roots, culture, and cuisine.

This current study will help address the identified gap in interventions by applying design thinking to culinary medicine approaches including creation of this prototype cookbook, and gathering formative implementation data. Here, we explore the design and piloting of a culturally-tailored cookbook intervention aimed at helping Filipino Americans make modifications in certain less healthy aspects of Filipino foods, by incorporating more plant-based foods, reducing sodium in cooking, and applying other heart-healthy eating strategies, while preserving attention to cultural cuisine and flavor. An additional goal is to empower individuals with hypertension to improve their blood pressure control through dietary change, ultimately helping to reduce the disproportionately high rates of hypertension that exist amongst Filipino Americans.

## 2. Methods: intervention design and pilot study

### 2.1. Human subjects ethics statement

This pilot study was added as an amendment onto a larger IRB approved study, “Comparative Effectiveness Study of Technologies to Promote Blood Pressure Control” conducted by Dr. Susan L. Ivey (PI). After discussion with The Committee for Protection of Human Subjects (CPHS), at the University of California, Berkeley, this pilot study did not require further approval from CPHS to proceed (IRB Protocol 2019-04-12136).

### 2.2. Formative work: applying design thinking

In this phase of the research project, Pack and team used an iterative design process (39) and culinary medicine approaches to create a prototype heart-healthy Filipino recipe book (going forward, we refer to this as the cookbook). First, the decision to use a recipe-based intervention was rooted in the culinary medicine principles of reducing the food knowledge gap (40), increasing development of food agency (41), and encouraging healthy food enjoyment (in replacement of enforcing unhealthy food avoidance) (33), through providing healthy ingredient choices, flavoring techniques, and cooking methods in these recipes (42). A key culinary medicine recommendation is to seek collaboration between nutrition experts, Registered Dietitians (RD), and specialists with culinary training (42). Thus, the first step was to engage several Filipino culinary experts from whom to seek advice, while also assembling a team of advisors including fellow students, a physician faculty expert in hypertension and lifestyle change, and an RD, who would provide input on design, development of recipes, dietary analysis of dishes based on average portion size, and basic dietary cardiovascular risk reduction principles for lowering blood pressure.

The team examined possible ways to make modifications to classic Filipino recipes, exploring potential ingredient substitutes, alternative cooking methods, and plant-based alternatives. The ultimate goal was to develop and/or identify existing recipes that would be heart-healthy, defined as lower sodium, lower fat, lower cholesterol, and higher fiber foods, while still being flavorful and culturally acceptable.

After networking and connecting with Filipino culinary experts in this field, Pack and team chose a diverse group of five Filipino individuals to contribute recipes and unique stories to the cookbook. In the selection process of choosing the five Filipino individuals to contribute to the cookbook, we carefully selected individuals who would add different perspectives on various themes that the team ultimately wanted to convey throughout the cookbook. There were no specific inclusion/exclusion criteria other than that the individuals had to identify as culturally Filipino and/or Filipino American and be in a “culinary and/or food related” career. The definition of a “culinary and/or food related” career was not strict, and ultimately diverse careers were represented in the individuals that were selected ranging from commercial chefs, herbalists, food justice advocates, business owners, and public health advocates. Ultimately, Pack and team reached out to five individuals to contribute to the cookbook through email and all five accepted to be interviewed and agreed to contribute a recipe to the cookbook.

In order to gather stories, each contributing individual met with Pack virtually through Zoom and was asked a subset of semi-structured interview questions pertaining to topics curated based on three major themes: (1) Topics focused on deconstructing and reversing the damaging narratives that portray Filipino cuisine as “unhealthy,” (2) Topics highlighting the importance of reconnecting with the land and foodways of our ancestors, and (3) Questions highlighting how to make healthy modifications to the Filipino cuisine, without compromising flavor profile. Questions for each contributing individual were tailored to the interviewee’s background and expertise. Interviews lasted around 30 min, were recorded, and subsequently transcribed. Each contributing individual was asked to donate a recipe to the cookbook and to describe their inspiration for the specific recipe they donated.

Once recipes were submitted by each culinary expert, an RD conducted nutrient analyses to evaluate the nutritional quality of each recipe and determined if they could be categorized as heart-healthy. To do this, the RD utilized ESHA Research’s Food Processor Nutrition Analysis software, which contained a food and nutrition database with information from the Food and Nutrient Database for Dietary Studies (FNDDS), the USDA Standard Reference database, manufacturer’s data, USDA FoodData Central Brands (43), among others, to create a nutrition label delineating the total calories, total fat, sodium, cholesterol, total carbohydrates, protein, vitamin D, calcium, iron, and potassium contained in a set portion of each recipe (as determined by the culinary expert). Based on this analysis, the RD ensured that each recipe was appropriate for people with cardiometabolic disease, and suggested heart-healthy modifications for recipes deemed to be out of specification. Specific nutrients focused on were total calories, total fat, and sodium, with the RD providing recommendations of decreasing serving size, decreasing ingredient amount, and replacing certain ingredients with healthier ones. Examples of these included recommending intake of a smaller portion size, decreasing the amount of sugar added, and utilizing low sodium soy sauce or light coconut milk instead of their calorically dense counterparts. Once adjustments were implemented and approved by the RD, an official nutrition label was created to be easily displayed and interpreted by cookbook readers.

### 2.3. Intervention design

**FIGURE 1:**
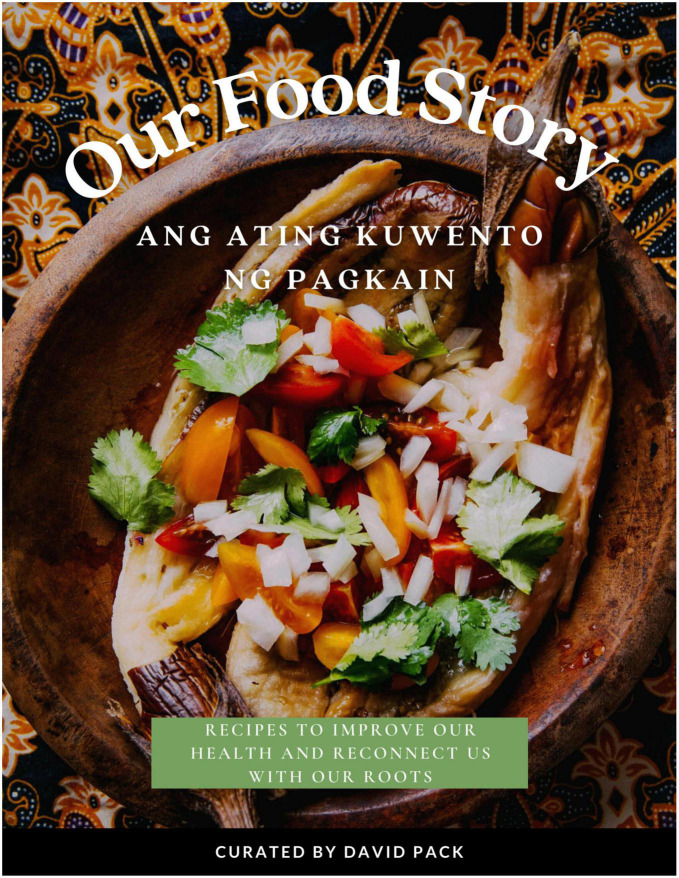
Introductory page of the cookbook, containing a floral and plant-based background. Extends an invitation to readers to start their culinary journey while reading the text.

**FIGURE 2:**
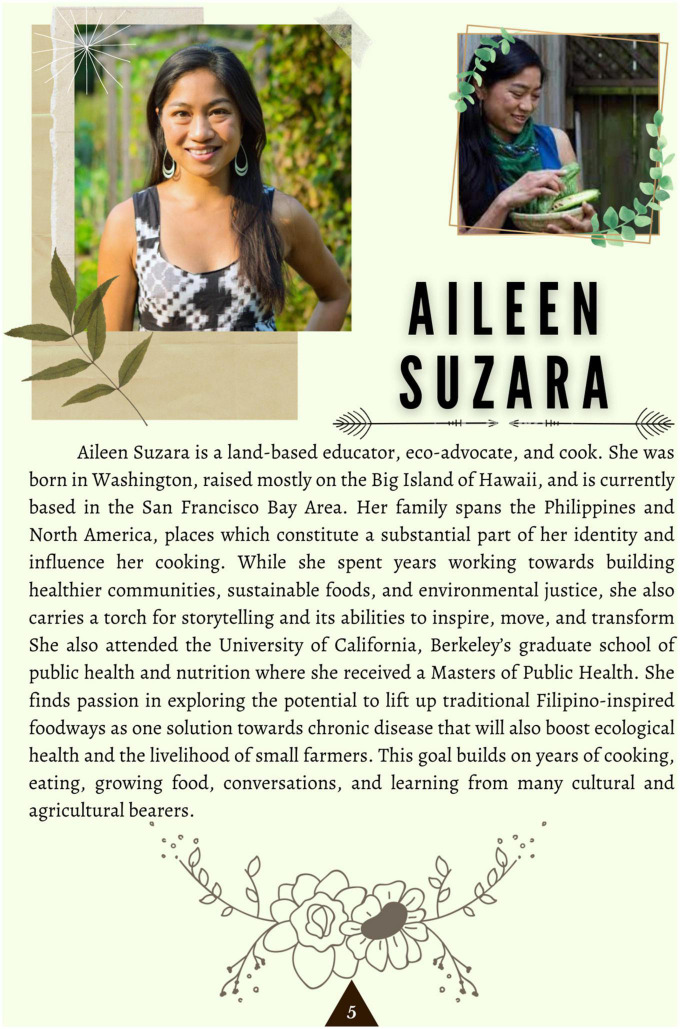
Culinary expert introduction and recipe forward, providing readers with the cultural background of the dish and importance of its heart-healthy modifications.

**FIGURE 3:**
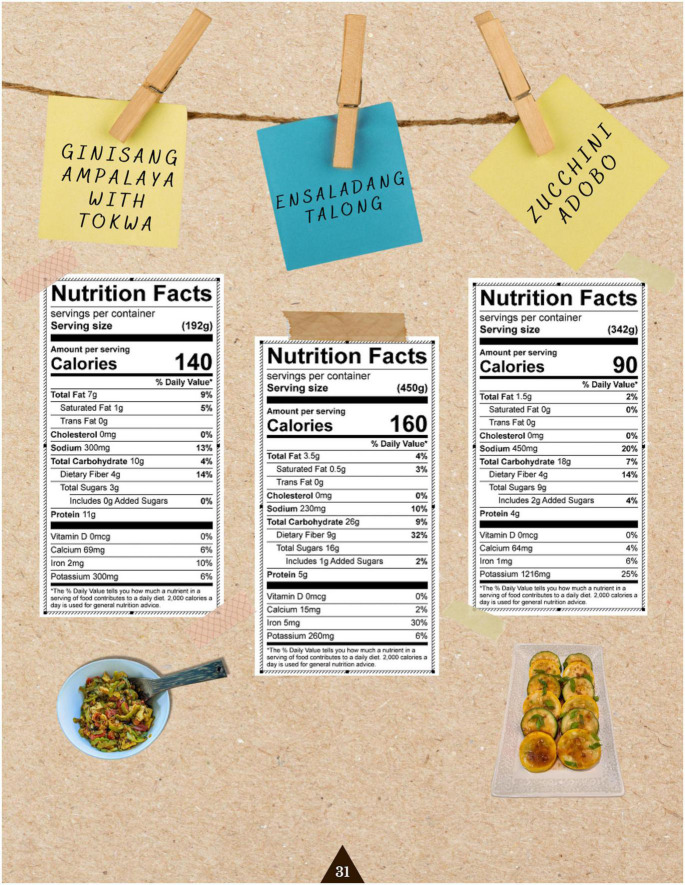
Nutrition labels of three recipes created by a Registered Dietitian. The page was formatted with images of the dish and casual design elements to be more approachable and understandable for our readers.

**FIGURE 4:**
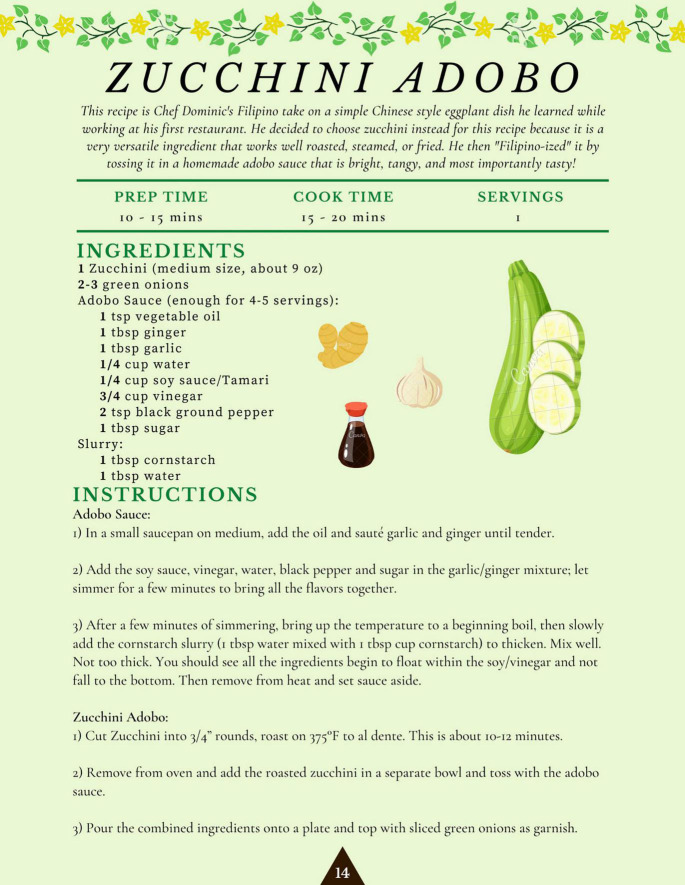
Zucchini adobo recipe featuring traditional ingredients and their accompanying graphics, as published in the cookbook.

The team then applied design thinking to structure the contents of the cookbook. Design thinking is an “applied research and innovation framework that prioritizes *empathy* for users of a service or product, involves highly diverse and collaborative project teams, and encourages an action-oriented rapid prototyping of user-derived insights rather than top down hypotheses” (44). This process involves five key components: the Empathize mode (observing and learning about the population of interest), the Define mode (framing a specific meaningful problem to address), the Ideate mode (collaborating with key stakeholders and brainstorming ideas to resolve the issue), the Prototype mode (creating tangible prototypes users can interact with), and the Test mode (refining prototypes through user feedback) (45). Applying this framework to our project, this prototype stage consisted of developing, testing out, and photographing the recipes, then compiling the text (including stories and chef information) and photos into a visually appealing, cohesive cookbook. This cookbook was created in Canva under a Pro Content License. The cookbook was designed with themes of tradition and “reconnecting to our roots” in mind, and with a goal of making each recipe inviting and less daunting to follow. This was conveyed through culturally relevant textiles and colored backgrounds, as well as floral and plant-based imagery applied throughout each page to evoke a sense of authenticity and familiarity (Figure 1). Complementary colors, ingredient graphics, and images of each recipe provided an enticing representation of each dish and demonstrated its feasibility to a proposed target audience. These recipes’ authenticity was supplemented with a foreword by each culinary expert, explaining their background, cultural significance of each dish, and rationale of their ingredient choices (Figure 2). In addition to the recipes, specific dietary education on the health benefits of each dish was included and written in a manner that was likely to be understandable to our population of interest. The nutrition facts of each recipe were similarly positioned to be more readable and less complex, through continued use of an inviting background, supporting graphics, and clear labeling (Figure 3). Overall, we created our cookbook with a story-telling framework, incorporated anecdotes and imagery, and paired recipes with health information to create a culturally competent heart-healthy Filipino cookbook. The prototype cookbook was reviewed by chefs, dietitians, and team members, and assessed to be ready to test out in a sample population. In addition to featuring classic Filipino recipes, priority was also placed on utilizing traditional Filipino ingredients, such as tamari, garlic, calamansi, coconut vinegar, and spicy peppers, and employing culturally-appropriate terminology throughout the cookbook, like *ampalaya* (in lieu of their westernized counterpart of bitter melon) and *patis* (fish sauce), paired with graphics that highlighted these ingredients (Figure 4).

### 2.4. Pilot study phase

Eligibility criteria for the pilot study included being over the age of 18, self-identifying as Filipino/a/x, self-reporting physician-diagnosed high blood pressure, and living in the San Francisco Bay Area. In order to screen for physician-diagnosed high blood pressure, potential participants were asked the question, “Have you previously been told by a physician that you have high blood pressure?”. The sampling frame for recruitment included community members drawn from local community free clinics, cultural-based organizations, and Filipino-serving community-based organizations. Ultimately, recruitment was done through presentations to these various organizations, virtual flyering, and through word-of-mouth. The recruitment process took place June 7th–June 18th, 2021. Once we developed the intervention, the team set a target goal to enroll at least 20 participants into the pilot study. We chose this target sample size of 20 participants due to the time and budget constraints of this small pilot study. A total of 22 participants were initially screened; however, two participants did not meet the eligibility criteria. Thus, twenty people who met the eligibility criteria were ultimately enrolled into the pilot study. We then asked the enrolled participants to use a copy of the cookbook prototype for 3 weeks, to cook at least one of the recipes in that period, and to complete a pre- and post-use survey including providing feedback on use of the recipes and cookbook, ease of cooking different dishes, and acceptability of the recipes.

Dietary change was assessed in the post-intervention survey through questions pertaining to fruit and vegetable consumption, sodium intake, and likelihood of using specified recommended actions to lower blood pressure, as well as through direct questions related to perceived motivation and success in achieving these dietary change behaviors.

### 2.5. Data collection

Participant data were gathered through structured surveys conducted using Qualtrics (Qualtrics, Provo, UT, USA) between June 19th and July 21st, 2021, for the baseline survey and August 4th–August 15th, 2021, for the post-intervention survey. The baseline survey was centered around topics of age, gender, educational attainment, living situation, self-rated general health status, fruit and vegetable intake, access to fresh and affordable foods, blood pressure medication use, COVID-19 impact, and contained questions from the Self-Care of Hypertension Inventory scale (SC-HI) (v. 2.0, March 2016) related to maintenance, confidence, and management for persons with hypertension, to obtain an understanding of their current health behaviors (46). This SC-HI scale instrument underwent content and scale validation in a sample of hypertensive individuals drawn from multiple outpatient clinical settings across the United States. This sample was gender and ethnically diverse (70% female, 60% Black, 32% White, and 6% Latino) with a mean age of 56.4 ± 13 years (46). Comparatively, our sample was 45% female, the mean age is 44.5 ± 14.5 years, and our sample was ethnically Filipino. The physician on the team (S. L. Ivey) had used these scales successfully in a local California population of diverse patients (∼20% API) with high blood pressure. The authors of the scale we used have successfully used this scale in Brazilian populations (47, 48) and have worked on a hypertension self-care scale in Filipino-Americans (49).

The post-intervention survey contained a mixture of closed and open-ended questions and was focused on dietary behavior change and usability and acceptability of the cookbook, with the first block containing questions about perceived health status, blood pressure control, motivation and success in dietary change, and questions from the Self-Care Management subscale from the SC-HI. The other two subscales of the SC-HI, the Self-Care Maintenance subscale and Self-Care Monitoring subscale, were not part of this survey as this intervention focused on promoting hypertension management behaviors, and we sought to increase participant engagement on this survey by using the survey instrument most relevant to our study. Given that each subscale is intended to be scaled and standardized separately, as per scale instructions provided by its authors, and noted to have good internal consistency (46), the removal of these two subscales from the survey does not impact the overall interpretation of participants’ hypertension management behaviors (50). The second block contained questions specifically related to the cookbook, with closed-ended questions about the ease of following recipes, affordability of ingredients, and their likelihood of recommending this cookbook to others. This block also utilized six open-ended questions that asked participants to share their opinions on each section of the cookbook–the story-telling approach, the text sections before and after each recipe, and the illustrations included–as well as their general thoughts on their experience using the cookbook, favorite aspects of the cookbook, and suggestions for improving it. All instruments and questions were in English, and interviews were conducted in English.

### 2.6. Data analysis: quantitative and qualitative

After the implementation phase, twenty pilot participants completed an exit survey to share their thoughts on the cookbook. In our analysis of the data, we compared participants’ closed-ended responses related to dietary and lifestyle behaviors before and after the 3 weeks period to evaluate changes in these factors that could be attributed to the intervention. These direct comparisons were made possible through the verbatim utilization of the same behavioral questions in both the pre- and post-intervention surveys.

First, we checked the normality of participants’ quantitative scaled scores through the Shapiro-Wilk normality test utilizing RStudio (version 4.2.2), to determine if a parametric test could be used to compare for significant differences in score before and after intervention. As this test resulted in a non-significant *p*-value (*p* > 0.05), we proceeded with a paired T-test to assess individuals’ scores for differences in behaviors. For Likert scale-based items, we conducted a proportion-based analysis to understand if the overall intervention group had shifted their perspectives toward specific aspects of hypertension-based behaviors. A Pearson’s chi-square test was conducted in RStudio to understand whether these shifts in proportion of each response were statistically significant. A proportion-based analysis was also conducted for qualitative, closed-ended responses in the post-intervention period that did not have a comparison counterpart in the pre-intervention survey, to understand attitudes of participants toward components of the cookbook. Graphical representations were created using RStudio for clearer presentation of this data.

To conduct qualitative data analysis, we utilized participants’ open-ended responses, in which participants disclosed how they felt toward the recipes, the design, the excerpts from the culinary experts, and any other suggestions or closing thoughts. These responses were evaluated and divided into these related categories: participant acceptability and satisfaction with intervention, and hypertension-based behavior changes following intervention. Two designated study team members worked together on this content analysis with thematic coding of participant responses, then the entire study team reviewed these categorizations and contributed to their interpretation. No inter-rater reliability was calculated given the very small number of interviews (*n* = 20). Through the categorization and analysis of responses, in conjunction with data from the quantitative analysis, we were able to organize interpretation of results.

## 3. Results

We first cover our qualitative results from the formative interviews, followed by results from the pilot participants.

### 3.1. Results of culinary expert interviews

Our team conducted formative individual interviews with the Filipino culinary experts who each provided a recipe for the cookbook. In these interviews, they shared their thoughts on the topics of authenticity, home, tradition, health, and inspiration for their recipes. We include exemplary quotes from the culinary experts here, illustrating their approach to making modifications to recipes in order to create lower sodium, plant-based, or higher fiber dishes – all examples of changes that might reduce cardiovascular risks.

When asked about what authentic Filipino food means to them, many of the culinary experts shared similar sentiments that Filipino food is ever evolving and cannot be constrained by a singular concept of what “real” Filipino food is. As one culinary chef noted:

*Authenticity changes. Food culture is not frozen in time, it changes. It’s not static. We need to leave room for change and for growth. We need to look back at the roots, and honor them, (but) also know that the ideas of (what is) authentic will shift, that we are part of making culture.*

This idea is reflected upon further by two other culinary experts, who provided the example of adobo, the national dish of the Philippines. One culinary expert explained how different regions of the Philippines may use different techniques and ingredients to create the same dish depending on what is readily available to them, yet both are still adobo. Another expert described how adobo was once considered not authentic since it popularly includes soy sauce, an ingredient with Chinese origins, but now adobo is the national dish. She went on to bring up other Filipino dishes that are Chinese influenced, such as pancit, siopao, lumpia, and how these are now indigenized. She also stated what she is trying to capture with her recipes:

*I am trying to recreate Filipino classics with plant-based ingredients. It is trying to capture the essence. It is not about re-writing the narrative.(They might feel) my nostalgia is not there because (I) changed the dish. But then in the Philippines, I realized the more different the better. They actually celebrate. “They put cheese in their pancit! Let’s go there.” But in the US, we feel like “how dare you put cheese in the pancit.” All I can do is have the best intentions. I hope they understand that I veganize with high respect. It boils down to intention and execution.*

Here, we gain insight into the approach of this culinary expert for creating plant-based Filipino dishes while retaining authenticity. She acknowledged that some may not find vegan Filipino food to be authentic, but believes that they are not questioning its authenticity, but rather what is personal and familiar to them. Knowing this, she highlighted the importance of being intentional with her recipes as well as embracing a non-static attitude toward authenticity. In her words, *“saying that Filipino food can only be made one way is going against the grain of what Filipino food is*.”

On the topic of health and how it relates to Filipino cuisine, one culinary expert discussed how “*the word ‘healthy’ can be charged*,” as it “*depends on everyone’s relationship to cooking, their class, our ancestral lineage, what was passed down to us, both the trauma and resilience.*” Given that in many communities, foods considered “*healthy*” may not be easily available or accessible, the concept of “*health*” may “*create some type of ableism*.” She explained that “*health does not have to be a binary construct or provoke polarization*,” and suggested, for example, that rather than directly asking people to remove meat from their diet, to ask them to add more vegetables. Many of the culinary experts emphasized that the association between food and health is complex and not one-dimensional, paralleling their attitudes toward authenticity.

As another culinary expert recounted:

*Food nourishes us in more than one way. Food nourishes us physically but also mentally, emotionally, and spiritually. Our traditional foods are ways in which we can illustrate those connections. Without these dishes, without access to these dishes, without access to ingredients, it also has a corollary effect on our overall health, not just physically but emotionally, mentally, and spiritually. There is a direct connection there.*

This culinary expert explained how food is related to various aspects of health and how the food we consume represents and embodies our relationships. He later shared how the recipe he provided was his lola’s (grandmother’s) recipe, and likely passed down by her lola before her, *“as a way to pass on that generational wisdom around how our foods are healing. It comes from our relationship with one another, our relationship with the earth, and our relationship with our ancestors and our culture*,” encapsulating his beliefs in the direct connection between food and our relationships.

These interviews provided valuable insight into the approaches of the project’s culinary experts for developing their recipes. We also included their words throughout the cookbook in order to provide these personal stories about their connection to food, ancestral roots, and how to make changes with respect for authenticity so readers might follow along during their culinary journey. Overall, the interviews with the culinary experts effectively allowed each of them to provide more details about their backgrounds and experiences, and how that has translated into their attitudes toward these crucial topics surrounding food and nutrition.

### 3.2. Results: participant pre- and post-intervention surveys (closed and open-ended results)

Here we detail the participants’ results following use of the cookbooks. All except two participants (of 20) noted they used one or more recipes, indicating a 90% participation rate.

#### 3.2.1. Sample characteristics and baseline behaviors

The sociodemographic characteristics of the participants (*n* = 20) are presented in Table 1. Among participants, 10 identified as male, nine identified as female, and one identified as non-binary; their age ranged from 23 to 71 years old (mean = 44.5). The majority of participants had obtained a degree from a 4 years college or university or higher (80%). While all participants spoke both Tagalog and English, a majority of participants identified English as their primary language spoken (75%). There was an even proportion of participants who were born in the US (50%) and not born in the US (50%), with a mean number of years living in the US of 40.4 years (22.0–52.0). No dropouts occurred across this 3 weeks intervention, with all twenty participants fully participating in the study and pre-post data collection.

**TABLE 1:** Sociodemographic characteristics of participants.

	Overall (*N* = 20)
**Age**	
Mean (SD)	44.5 (14.5)
Median (min, max)	42.5 (23.0, 71.0)
**Gender**	
Female	9 (45.0%)
Male	10 (50.0%)
Non-binary	1 (5.0%)
**Living alone?**	
No	17 (85.0%)
Yes	3 (15.0%)
**Currently working at a job?**	
No	7 (35.0%)
Yes	13 (65.0%)
**Providing care work?**	
No	12 (60.0%)
Yes	8 (40.0%)
**Unemployment**	
Refused	1 (5.0%)
No	16 (80.0%)
Yes	3 (15.0%)
**Currently searching for work?**	
No	18 (90.0%)
Yes	2 (10.0%)
**Number of hours working at a job or providing care work**	
Mean (SD)	48.6 (18.7)
Missing	6 (30.0%)
**Number of primary care visits (12 months)**	
Mean (SD)	1.40 (0.940)
**Number of years living in the US**	
Mean (SD)	40.4 (11.8)
Missing	10 (50.0%)
**Primary language spoken**	
English	15 (75.0%)
Tagalog	5 (25.0%)
**Education level**	
High school or lower	2 (10.0%)
2 years junior or community college	2 (10.0%)
4 years college or university	12 (60.0%)
Graduate or professional school	4 (20.0%)
**Born in the US?**	
No	10 (50.0%)
Yes	10 (50.0%)
**Number of times eating fruit per week**	
Mean (SD)	6.15 (5.45)
Median (min, max)	4.00 (0, 21.0)
**Number of times eating vegetables per week**	
Mean (SD)	7.71 (7.30)
Median (min, max)	5.00 (0, 28.0)
Missing	1 (5.0%)
**How often fruits and vegetables are affordable**	
Always	7 (35.0%)
Sometimes	2 (10.0%)
Usually	11 (55.0%)
Never	0 (0%)
**How often fruits and vegetables are accessible**	
Always	14 (70.0%)
Sometimes	0 (0%)
Usually	6 (30.0%)
Never	0 (0%)
**High blood pressure within the last month**	
Don’t know	8 (40.0%)
No	5 (25.0%)
Yes	7 (35.0%)
**Currently taking blood pressure medication**	
Don’t know	1 (5.0%)
No	10 (50.0%)
Yes	9 (45.0%)
**Had experienced lifestyle changes due to COVID-19**	
No	8 (40.0%)
Yes	12 (60.0%)
**Eating habits changes due to COVID-19**	
Don’t know	2 (10.0%)
Much more healthy	7 (35.0%)
Somewhat more healthy	8 (40.0%)
Somewhat less healthy	3 (15.0%)
Much less healthy	0 (0%)
**Hypertension self-care management scaled score (%)**	
Mean (SD)	63.8 (22.7)
Median (min, max)	66.7 (8.33, 91.7)
**Hypertension self-care maintenance scaled score (%)**	
Mean (SD)	50.9 (13.2)
Median (min, max)	51.5 (15.2, 75.8)
Missing	1 (5.0%)
**Hypertension self-care confidence scaled score (%)**	
Mean (SD)	60.8 (17.9)
Median (min, max)	61.1 (27.8, 94.4)
Missing	1 (5.0%)

At baseline, participants reported consuming fruits and vegetables an average of 2.76 and 3.53 times per week, respectively. Adequate access and affordability of fruits and vegetables in their current food environments were observed, with 70% reporting that they always could find fruits and vegetables and 30% reporting that they could usually find them, as well as 35% reporting that they were always affordable, 55% reporting that they were usually affordable, and 10% reporting that they were sometimes affordable. The COVID-19 pandemic was also perceived to cause lifestyle changes among a majority of participants (60%), with 35% reporting that their eating habits became much healthier, 40% reporting that they were somewhat more healthy, and 15% reporting that they were somewhat less healthy.

When assessing hypertensive self-care behaviors through the three SC-HI sub-scales at baseline, participants had an average hypertension management score of 63.8%, maintenance score of 50.9%, and confidence score of 60.8%, indicating moderate levels of self-care.

#### 3.2.2. Participant acceptability and satisfaction with intervention

Participants expressed that they highly resonate with the level of detail incorporated in both the recipes and the explanations behind their cultural aspects by the culinary experts, as it brought forth shared feelings of connection and empathy that encouraged them to try these heart-healthy recipes. As one participant wrote, “*I really loved the story telling aspect of this cookbook because it gave deeper meaning and connection to the food*.” Another participant reciprocated this positive feeling of having an understanding of the goal of the cookbook and shared how this was a motivating factor in sparking dietary change: “*I understand your approach. Food is medicine. The flavor profile was definitely there. The stories, along with the pictures made it compelling enough for me to try the recipes. It’s a start. we all must have a beginning*.” It is evident that the intentional design of including the stories from the culinary experts had a beneficial impact on motivation levels for pilot participants and promoted familiarity with the recipes as participants tried them for the first time.

Furthermore, participants reported that the culturally tailored aspects of this cookbook helped shift their perspective on the feasibility of managing their hypertension by changing their dietary habits while still retaining their culture, with one participant describing their prior perception of being unable to feel healthy while eating Filipino foods: “*It felt nice to eat Filipino food and not feel very guilty about it*!” This optimistic sentiment of having a unique culturally-tailored invention was echoed by other participants, with another stating that “*Filipino cooking, as we know, is not the healthiest. But if there are recipes with healthier options available, we can continue to perpetuate our culture through food and feel good about ourselves at the same time.*” Such feedback from participants demonstrated how they understood and embraced a major goal of the cookbook and the culinary experts–to begin transforming fixed, negative thoughts on the healthiness of Filipino food by introducing a dynamic, multi-faceted, growth mindset toward health and food.

Thus, the inclusion of excerpts from these interviews into the cookbook enabled participants to obtain a deeper understanding of the stories and the roads traveled by the cookbook’s culinary experts to provide a more intimate and guided culinary experience. This design element empowered readers to embark on dietary change as they tried out these novel heart-healthy recipes themselves.

Overall, participant responses from the post-intervention surveys highlighted the practical applications of this cookbook and suggest the preliminary feasibility of circulating the cookbook to a wider audience. All participants reported a positive degree of likelihood of recommending this cookbook to someone else–85% of participants stated that they were “very likely” to recommend, 10% of participants reported they were “somewhat likely” to, and zero participants reported that they would not recommend it.

#### 3.2.3. Improvements in hypertensive self-care behaviors

While two of twenty participants reported they did not cook any recipes, all twenty participants were noted to have read through and thus understood the contents of the cookbook through their detailed open-ended responses. Furthermore, this high instruction compliance rate of 90% indicates that a majority of this group of Filipino American hypertensive individuals were willing to test one or more recipes.

**FIGURE 5:**
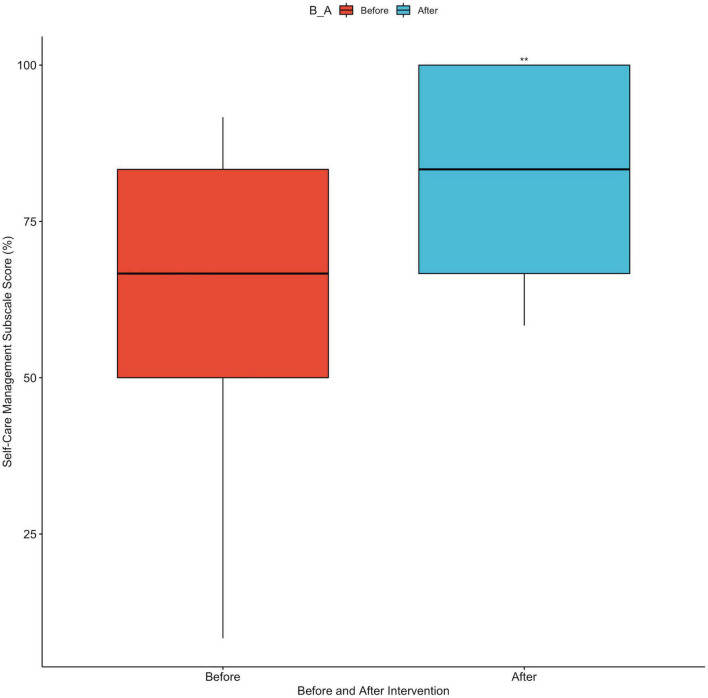
Boxplot of Self-Care Management subscale scores before and after intervention. **Indicates statistically significant difference, *p* < 0.008.

Comparing participants’ average Self-Care Management subscale scores from before ($\bar{x}$ = 63.75%) to after intervention ($\bar{x}$ = 80.83%) revealed an average increase of 17% across the study population (Figure 5). Assessing individuals’ scores for differences in behaviors yielded significant results (*p* < 0.008), indicating that each individual participant was more likely to implement recommended actions to reduce their blood pressure after using the cookbook for 3 weeks. This behavior change is supported by an increased proportion of participants reporting they always eat fruits and vegetables (10% increase), an increase in participants reporting that they eat a low salt diet daily or frequently (10% increase), and an increased proportion of participants reporting that they frequently ask for low salt items when eating outside of their household (5% increase) after completing the intervention, as seen in Table 2. Although increases are non-significant (*p* > 0.05), these positive changes across multiple behaviors essential for hypertensive lifestyle change indicate a trend to follow in future rigorous studies.

**TABLE 2:** Categorical sum of participant responses to selected Self-Care Maintenance subscale questions, before and after receiving intervention.

Self-Care Maintenance subscale questions		Response (%)				
		**Never or rarely**	**Sometimes**	**Frequently**	**Always or daily**	**Missing**
How routinely do you eat lots of fruits and vegetables?	Before	5	25	40	25	5
	After	0	30	35	35	0
	Net change	–5	+5	–5	+10	–5
How routinely do you eat a low salt diet?	Before	10	75	10	5	0
	After	25	50	15	10	0
	Net change	+15	–15	+5	+5	0
How routinely do you ask for low salt items when eating out or visiting others?	Before	65	30	5	0	0
	After	55	35	10	0	0
	Net change	–10	+5	+5	0	0

**FIGURE 6:**
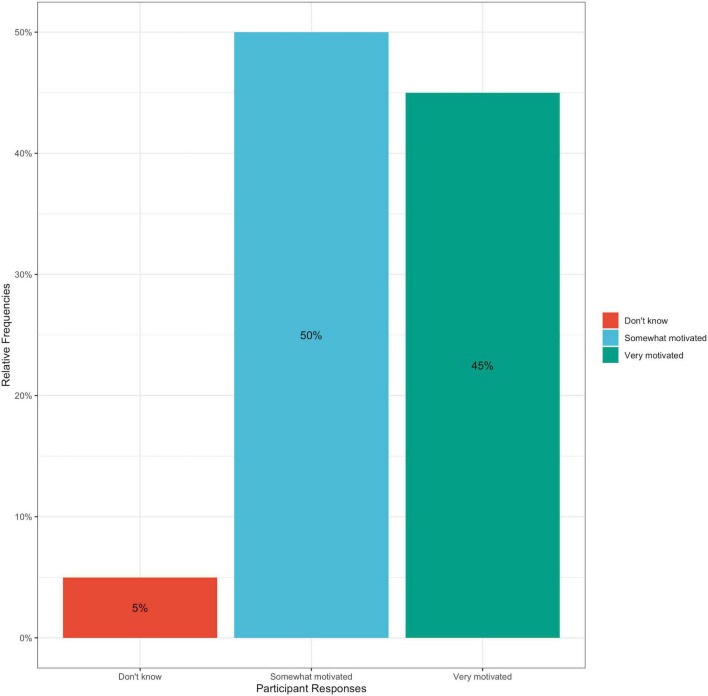
Bar graph of percentage of participant responses to post-intervention survey question: How motivated would you say you have felt during the past 3 weeks to achieve dietary change? Answer choices included: Very motivated, somewhat motivated, neither, somewhat unmotivated, and very unmotivated.

**FIGURE 7:**
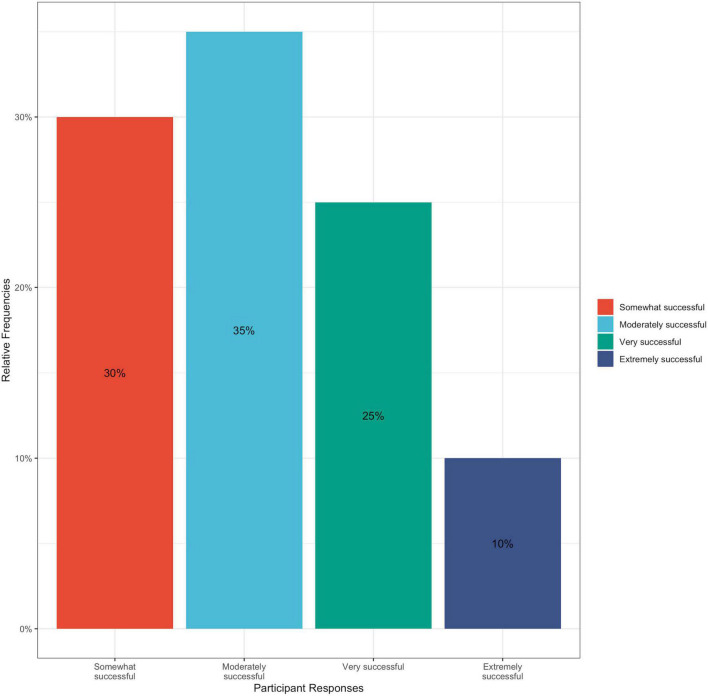
Bar graph of percentage of participant responses to post-intervention survey question: Thinking about the past 3 weeks, how successful would you say you have been in achieving dietary change? Answer choices included: Extremely successful, very successful, moderately successful, somewhat successful, and not at all successful.

**FIGURE 8:**
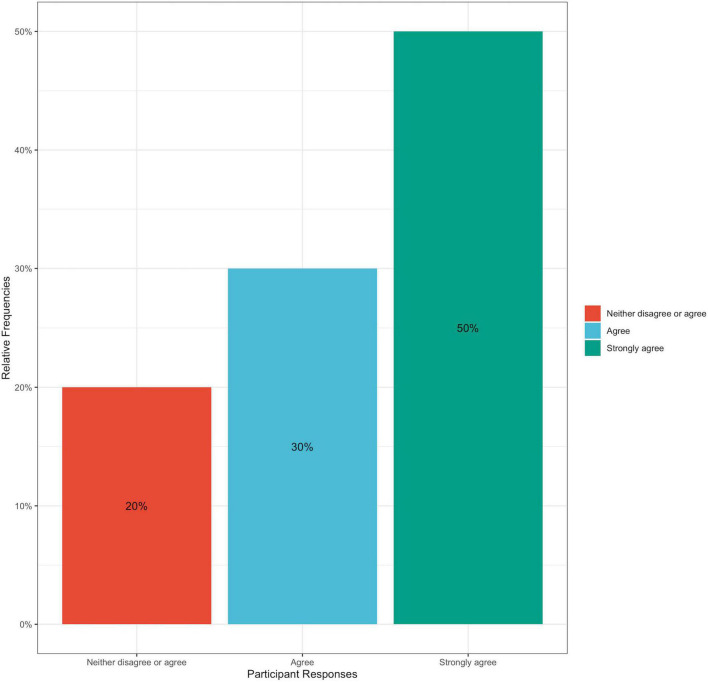
Bar graph of percentage of participant responses to post-intervention survey question: How much do you agree that the following statement is true for you: “I intend to continue to work to achieve dietary behavior change to improve my blood pressure”? Answer choices included: Strongly agree, agree, neither agree or disagree, disagree, and strongly disagree.

This trend in improving hypertensive self-care behavior change was also observed in participants when they were asked to reflect on their motivation to achieve dietary change during their 3 weeks with the cookbook; 95% of participants responded that they felt at least somewhat motivated to achieve dietary change, and no participants reported being “*unmotivated*” (Figure 6). All participants reported varying levels of success in achieving dietary change within their 3 weeks of using the cookbook, with 10% reporting that they were extremely successful, 25% reporting that they were very successful, 35% reporting moderate success, and 30% reporting that they were somewhat successful (Figure 7). This cookbook was also seen to inspire further dietary change, with 80% of participants agreeing or strongly agreeing with the statement “*I intend to continue to work to achieve dietary behavior change to improve my blood pressure*” (Figure 8). These results are summarized in Table 3.

**TABLE 3:** Participant responses to post-intervention survey questions related to dietary behavior change.

Post-intervention survey questions	Likert scale responses (%)						
	**1**	**2**	**3**	**4**	**5**	**Don’t know**	**Refused**
How motivated would you say you have felt during the past 3 weeks to achieve dietary change? You can answer: “Very unmotivated” (1), “Somewhat unmotivated” (2), “Neither” (3), “Somewhat motivated” (4), or “Very motivated” (5)	0	0	0	50	45	5	0
Thinking about the past 3 weeks, how successful would you say you have been in achieving dietary change? You can answer: “Not at all successful” (1), “Somewhat successful” (2), “Moderately successful” (3), “Very successful” (4), or “Extremely successful” (5)	0	30	35	25	10	0	0
How much do you agree that the following statement is true for you: “I intend to continue to work to achieve dietary behavior change to improve my blood pressure” You can answer: “Strongly disagree” (1), “Disagree” (2), “Neither disagree or agree” (3), “Agree” (4), or “Strongly agree” (5)	0	0	20	30	50	0	0

Participants also expressed appreciation for how the recipes were tasty, easy to follow, and overall appealing. This valuable feedback indicates the cookbook was well-received by participants, suggesting the practicality of distributing this intervention in a larger, more diverse population. Participants’ open-ended answers about their experiences during the intervention phase further supports this idea, with one participant stating: “*The recipes were accessible and not very daunting.*” Another participant previously believed making heart-healthy dietary changes equated to a trade-off with taste, but shares how this cookbook helped transform their attitude: “*The recipes were easy to follow and tasteful even when the recipes only ask for a small portion of salt, oil, etc. They were delicious.*” Participants also attributed the effectiveness of this cookbook to its design elements, with its illustrations and photographs playing a large part in both understanding the recipe and motivating them to use the recipes. A participant explicitly pointed out that the images “*made me want to try out the recipes*,” while another described their thoughts on the functionality of the design:

*I thought the illustrations and pictures were a nice addition and a necessity with any recipe book. Some people may not be familiar with some of the vegetables featured in the recipes, plus it gives us a guide on how the dishes are supposed to look like, at the end.*

Overall, the design thinking approach used to create this cookbook, paired with the comprehensibility of materials fostered by the culinary experts and dietitian expert, were observed to encourage positive health behavior change, further emphasizing the need for incorporation of culturally tailored dietary/cooking instruction into hypertension-based interventions.

Although participants’ blood pressures were not measured in this pilot study, the exit survey revealed participants reported lower perceived blood pressure post-intervention. Specifically, 71% of participants who stated their blood pressure was high during the month prior to the study, reported their blood pressure was not high within the month of receiving and using the cookbook. This finding points to the preliminarily positive physiological effects of this intervention, which could be investigated in a larger study with a more rigorous design.

## 4. Discussion

This research project included a design phase paired with a small feasibility pilot assessing the effects of a unique culturally-tailored cookbook on dietary changes with hypertensive Filipino Americans. Using design thinking principles, the team worked with culinary experts, chefs, and advisors to create a cookbook that would reach our population of interest. The cookbook integrated storytelling with cultural recipes to better engage this population. The cookbook also featured selected information in Tagalog. The recipes were created in partnership with culinary experts and chefs to incorporate more plant-based foods and reduce sodium in traditional Filipino American dishes while retaining preferred flavors. This study demonstrated that the tailored cookbook was well-accepted by the population of interest and was effective in encouraging dietary change, with a future goal of reducing blood pressure.

The input received from culinary experts, paired with positive acceptance and behavior change reported by participants, provides a starting point for further hypertension intervention developments in the space of culturally-sensitive approaches for this underserved population. While existing literature highlights the disproportionate rate at which Filipino Americans are impacted by hypertension (3, 4, 6, 7), the lack of disaggregated data (3), and minimally available culturally-specific studies (22) conducted on this subpopulation underscores the necessity of this pilot study. Furthermore, multiple studies conducted on understanding health promotion beliefs and attitudes in the Filipino American population have emphasized the importance of incorporating these cultural considerations into healthcare practices and recommendations (9, 11, 15, 16, 20), as this formative study is focused on developing. Published studies focused on the Filipino American population that have utilized these cultural factors in their interventions have only included diet as an aspect of a multi-component hypertension-based intervention (22) or have only focused their interventions on non-hypertension conditions (23, 24). The results of our study also align with results observed in other culinary medicine-based studies, with the increase in self-efficacy, improved dietary attitudes, and improved dietary behavior change reported in literature (32, 34), as well as a significant increase in participants’ Self-Care Management scores, following our culinary medicine intervention. Thus, this study addresses the lack of robust literature on culturally-nuanced dietary approaches developed to reduce hypertension in the Filipino American population and demonstrates the appeal of specifically tailored interventions for high risk groups.

### 4.1. Strengths

Strengths of this study include extensive front end processes to connect with Filipino American culinary experts, who would have interest and capability to provide heart-healthy recipes, and a student design team with caring connections to heritage and visual design that would appeal to the target audience, as well as a registered dietitian who volunteered time to conduct nutritional analyses. While the pilot is small, by engaging with a local Filipino-serving community clinic, we were able to recruit individuals who reported a prior diagnosis of hypertension. First author (D. Pack) also brought “in language” resources to translate sections into Tagalog, given this was a very low budget student-driven project of the heart. This is one of just a handful of studies conducting research pertaining to cardiometabolic-based interventions for Filipinos, with only one published paper to date investigating culturally-relevant dietary modifications for this large, underserved subset of the population. Furthermore, the results of this study corroborate the positive dietary change findings observed in culinary medicine interventions conducted in other populations, indicating a high likelihood of success in applicability to hypertension. Thus, the novel approach of this study, focused on culturally-tailored culinary medicine, will help fill gaps in the literature and greatly augment this field of study.

### 4.2. Limitations

Limitations of this study included recruiting challenges, small sample size, and challenges finding resources to scale the intervention. Participants were recruited through snowballing and community flyering. These methods were restricted in reach and may bias who participates. While this was a pilot study, the small sample size and lack of a control group impacts power to detect changes and generalizability of findings. This was a low budget student-driven project and lacked the personnel and funding to scale the intervention including inability to recruit a larger number of participants, and challenges in funding cookbook printing (estimated at $12.50/cookbook). Analytic approaches were confined to univariate and bivariate analyses of survey data by students.

Additionally, participants were provided with an electronic version of the cookbook, instead of being sent a physical copy. Given that this study population included older adults, a digital “only” format could lead to decreased usability of all elements of the cookbook and lower adherence to its comprehensive applications. Limited resources also led to constraints in language use, with the cookbook only utilizing Tagalog and English languages available to the student team, despite the diverse range of dialects spoken by the larger Filipino American community.

## 5. Conclusion and next steps

The post-intervention survey revealed additional insights on the usability of the cookbook, as well as the feasibility of implementing this unique intervention in the wider population. All but two of the twenty participants tried at least one recipe in the cookbook and many found the ingredients were accessible and affordable. Participants also expressed appreciation for how the recipes were tasty, easy to follow, and overall appealing. This valuable feedback indicates the cookbook was well-received by participants, suggesting the practicality of distributing this intervention in a larger, more diverse population. Participants’ open-ended answers about their experiences during the intervention phase further support this idea, with participants noting that recipes were easy to use, as well as that recipes they tried were found to be flavorful. Participants also enjoyed the design elements including illustrations and stories. These statements highlighted the importance of incorporating a design-thinking approach and cultural considerations in fostering motivation toward positive behavior change, which was also seen through significant improvements in each participant’s hypertension-based Self-Care Management score. Thus, this study contributes to existing literature on the importance of understanding health promotion beliefs in ethnic minority populations and prioritizing these cultural factors in best healthcare practices and providing recommendations. As this study is one of the first to examine a culturally-focused dietary approach as an exclusive intervention for hypertensive Filipino-Americans, this serves as a potential starting point for the expansion of robust literature on specific culturally-tailored dietary interventions for similar high risk groups.

In conclusion, this unique intervention applied design thinking and culturally imbued knowledge of cuisines to provide a culturally-tailored intervention that helped encourage dietary change in hypertensive Filipino Americans struggling to control and manage their blood pressure. While small in scale, the results achieved through this pilot study provided knowledge about feasibility, acceptability, and positive dietary change which serve as grounds for scaling up the study into a more robust, follow-up randomized controlled trial comparing measured blood pressure outcomes of a control vs. intervention group. In a subsequent follow-up study, a larger sample size would be recruited, blood pressure changes would be assessed by monitoring measured blood pressure before and after the intervention, and a longer study period would be used in order to better measure the impact of the intervention. In addition, to fully evaluate this cookbook’s impact on hypertensive behavior change, data related to potential external confounding factors would be measured at both baseline and post-intervention. Lastly, the creation of different cookbook versions in other Filipino language dialects would be beneficial to test in a larger sample size that encompasses participants from a wider variety of Filipino American subgroups. This culturally-tailored intervention was seen to be extremely meaningful to participants from the Filipino American community, a minoritized ethnic population. In addition, the positive changes observed in dietary behavior are promising. Such an intervention can change the landscape of dietary change for hypertensive individuals who seek a more diverse selection of culturally nuanced cuisines with heart health goals in mind.

## Data availability statement

The raw data supporting the conclusions of this article will be made available by the authors, without undue reservation.

## Ethics statement

The studies involving human participants were reviewed and approved by the Committee for Protection of Human Subjects (CPHS) at the University of California, Berkeley. The patients/participants provided their written informed consent to participate in this study. Written informed consent was obtained from the individual(s) for the publication of any potentially identifiable images or data included in this article.

## Author contributions

DP and SI contributed to the conception and design of the study. NY contributed to the design of the intervention. DP, MS, and AD performed the survey analysis. DP wrote the manuscript. SI edited the first draft of the manuscript. MS, DP, MV, NY, AD, and SI wrote sections of the final manuscript. All authors contributed to manuscript revision, read, and approved the submitted version.

## References

[1] U.S. Census Bureau. *Profile of General Demographic Characteristics.* Suitland, MD: U.S. Census Bureau (2020).

[2] Budiman A Ruiz NG. *Key Facts about Asian Origin Groups in the U.S.* Washington, DC: Pew Research Center (2022).

[3] Gordon NP Lin TY Rau J Lo JC. Aggregation of Asian-American subgroups masks meaningful differences in health and health risks among Asian ethnicities: an electronic health record based cohort study. *BMC Public Health.* (2019) 19:1551. 10.1186/s12889-019-7683-3 31760942 PMC6876105

[4] Adia AC Nazareno J Operario D Ponce NA. Health conditions, outcomes, and service access among Filipino, Vietnamese, Chinese, Japanese, and Korean adults in California, 2011–2017. *Am J Public Health.* (2020) 110:520–6. 10.2105/AJPH.2019.305523 32078359 PMC7067106

[5] Tsao CW Aday AW Almarzooq ZI Alonso A Beaton AZ Bittencourt MS Heart disease and stroke statistics—2022 update: a report from the American heart association. *Circulation.* (2022) 145:e153–639. 35078371 10.1161/CIR.0000000000001052

[6] Ma GX Lee M Bhimla A Tan Y Gadegbeku CA Yeh MC Risk assessment and prevention of hypertension in Filipino Americans. *J Community Health.* (2017) 42:797–805. 10.1007/s10900-017-0320-0 28161775 PMC5494270

[7] Sales C Lin B Palaniappan L. *Philippine and philippine-American health statistics, 1994–2018. Report No. 1*. (2020). Available online at: https://med.stanford.edu/content/dam/sm/care/PH-Data-Brief.pdf

[8] Ursua R Aguilar D Wyatt L Tandon SD Escondo K Rey M Awareness, treatment and control of hypertension among Filipino immigrants. *J Gen Intern Med.* (2014) 29:455–62. 10.1007/s11606-013-2629-4 24113806 PMC3930791

[9] Abesamis CJ Fruh S Hall H Lemley T Zlomke KR. Cardiovascular health of Filipinos in the United States: a review of the literature. *J Transcult Nurs.* (2016) 27:518–28. 10.1177/1043659615597040 26243715 PMC5595148

[10] Lara-Breitinger K. *Cardiovascular Disease in the Filipino American Community: Revisiting Our Beloved Filipino-Comfort Foods – The Early Career Voice.* (2022). Available online at: https://earlycareervoice.professional.heart.org/cardiovascular-disease-in-the-filipino-american-community-revisiting-our-beloved-filipino-comfort-foods/ (accessed January 30, 2023).

[11] Garcia GM Romero RA Maxwell AE. Correlates of Smoking Cessation Among Filipino Immigrant Men. *J Immigr Minor Health.* (2010) 12:259–62. 10.1007/s10903-009-9244-9 19296220 PMC2839465

[12] Batcagan-Abueg AP Lee JJ Chan P Rebello SA Amarra MS. salt intakes and salt reduction initiatives in Southeast Asia: a review. *Asia Pac J Clin Nutr.* (2013) 22:490–504. 24231008 10.6133/apjcn.2013.22.4.04

[13] Sievenpiper JL. Low-carbohydrate diets and cardiometabolic health: the importance of carbohydrate quality over quantity. *Nutr Rev.* (2020) 78(Suppl. 1):69–77. 10.1093/nutrit/nuz082 32728757 PMC7390653

[14] NIH. *DASH Eating Plan | NHLBI.* (2021). Available online at: https://www.nhlbi.nih.gov/education/dash-eating-plan (accessed January 31, 2023).

[15] Johnson-Kozlow M Matt GE Rock CL de la Rosa R Conway TL Romero RA. Assessment of dietary intakes of Filipino-Americans: implications for food frequency questionnaire design. *J Nutr Educ Behav.* (2011) 43:505–10. 10.1016/j.jneb.2010.09.001 21705276 PMC3204150

[16] Vanstone M Rewegan A Brundisini F Giacomini M Kandasamy S DeJean D. Diet modification challenges faced by marginalized and nonmarginalized adults with type 2 diabetes: a systematic review and qualitative meta-synthesis. *Chronic Illn.* (2017) 13:217–35. 10.1177/1742395316675024 27884930

[17] Mitchell JA Perry R. Disparities in patient-centered communication for Black and Latino men in the U.S.: cross-sectional results from the 2010 health and retirement study. *PLoS One.* (2020) 15:e0238356. 10.1371/journal.pone.0238356 32991624 PMC7523955

[18] Johnson RL Roter D Powe NR Cooper LA. Patient race/ethnicity and quality of patient–physician communication during medical visits. *Am J Public Health.* (2004) 94:2084–90. 10.2105/AJPH.94.12.2084 15569958 PMC1448596

[19] Charles-Alexis A. *Diversify Nutrition: The Need for Cultural Competence in Dietetics.* San Francisco, CA: Healthline (2021).

[20] Domingo JLB. Strategies to increase Filipino American participation in cardiovascular health promotion: a systematic review. *Prev Chronic Dis.* (2018) 15:170294. 10.5888/pcd15.170294 29786501 PMC5985898

[21] Nur PRMQ. Physician interaction and counseling of Filipino patients. *Proceedings of the Filipino American Cardiovascular Health Conference.* National Harbor, MD: (2011).

[22] Ma GX Bhimla A Zhu L Beeber M Aczon F Tan Y Development of an intervention to promote physical activity and reduce dietary sodium intake for preventing hypertension and chronic disease in Filipino Americans. *J Racial Ethn Health Disparit.* (2021) 8:283–92. 10.1007/s40615-020-00781-z 32495306 PMC7710586

[23] Dirige OV Carlson JA Alcaraz J Moy KL Rock CL Oades R Siglang Buhay: nutrition and physical activity promotion in Filipino-Americans through community organizations. *J Public Health Manag Pract.* (2013) 19:162. 10.1097/PHH.0b013e3182571708 23358295

[24] Leake AR Bermudo VC Jacob J Jacob MR Inouye J. Health is wealth: methods to improve attendance in a lifestyle intervention for a largely immigrant Filipino-American sample. *J Immigr Minor Health.* (2012) 14:475–80. 10.1007/s10903-011-9487-0 21647623

[25] Resnicow K Wallace DC Jackson A Digirolamo A Odom E Wang T Dietary change through African American churches: baseline results and program description of the eat for life trial. *J Cancer Educ.* (2000) 15:156–63. 11019764 10.1080/08858190009528685

[26] Brown DL Conley KM Resnicow K Murphy J Sánchez BN Cowdery JE Stroke health and risk education (SHARE): design, methods, and theoretical basis. *Contemp Clin Trials.* (2012) 33:721–9. 10.1016/j.cct.2012.02.020 22421317 PMC3361513

[27] Bianchi F Dorsel C Garnett E Aveyard P Jebb SA. Interventions targeting conscious determinants of human behaviour to reduce the demand for meat: a systematic review with qualitative comparative analysis. *Int J Behav Nutr Phys Act.* (2018) 15:102. 10.1186/s12966-018-0729-6 30340498 PMC6194670

[28] Ayala GX Pickrel JL Baquero B Sanchez-Flack J Lin SF Belch G The El Valor de Nuestra Salud clustered randomized controlled trial store-based intervention to promote fruit and vegetable purchasing and consumption. *Int J Behav Nutr Phys Act.* (2022) 19:19. 10.1186/s12966-021-01220-w 35177070 PMC8851758

[29] Sacks FM Svetkey LP Vollmer WM Appel LJ Bray GA Harsha D Effects on blood pressure of reduced dietary sodium and the dietary approaches to stop hypertension (DASH) Diet. *N Engl J Med.* (2001) 344:3–10. 10.1056/NEJM200101043440101 11136953

[30] Razavi AC Dyer A Jones M Sapin A Caraballo G Nace H Achieving dietary sodium recommendations and atherosclerotic cardiovascular disease prevention through culinary medicine education. *Nutrients.* (2020) 12:3632. 10.3390/nu12123632 33255901 PMC7761274

[31] La Puma J. What is culinary medicine and what does it do? *Popul Health Manag.* (2016) 19:1–3. 10.1089/pop.2015.0003 26035069 PMC4739343

[32] Asher RC Shrewsbury VA Bucher T Collins CE. Culinary medicine and culinary nutrition education for individuals with the capacity to influence health related behaviour change: a scoping review. *J Hum Nutr Diet.* (2022) 35:388–95. 10.1111/jhn.12944 34415642

[33] Irl BH Evert A Fleming A Gaudiani LM Guggenmos KJ Kaufer DI Culinary medicine: advancing a framework for healthier eating to improve chronic disease management and prevention. *Clin Ther.* (2019) 41:2184–98. 10.1016/j.clinthera.2019.08.009 31543284

[34] Hasan B Thompson WG Almasri J Wang Z Lakis S Prokop LJ The effect of culinary interventions (cooking classes) on dietary intake and behavioral change: a systematic review and evidence map. *BMC Nutr.* (2019) 5:29. 10.1186/s40795-019-0293-8 32153942 PMC7050805

[35] Tan FCJH Oka P Dambha-Miller H Tan NC. The association between self-efficacy and self-care in essential hypertension: a systematic review. *BMC Fam Pract.* (2021) 22:44. 10.1186/s12875-021-01391-2 33618661 PMC7901221

[36] Writing Group of the Premier Collaborative Research Group. Effects of comprehensive lifestyle modification on blood pressure controlmain results of the PREMIER clinical trial. *JAMA.* (2003) 289:2083–93. 10.1001/jama.289.16.2083 12709466

[37] Villalona S Ortiz V Castillo WJ Garcia Laumbach S. Cultural relevancy of culinary and nutritional medicine interventions: a scoping review. *Am J Lifestyle Med.* (2021) 16:663–71. 10.1177/15598276211006342 36389044 PMC9644144

[38] Group by Doctor’s Teaching Kitchen. *Culinary medicine Philippines [Internet]*. (2019). Available online at: https://www.facebook.com/groups/339173066956093/about (accessed April 6, 2023).

[39] Hasso Plattner Institute of Design at Stanford University. *More about Design Thinking — Stanford d.school.* (2019). Available online at: https://dschool.stanford.edu/executive-education-resource-collections/keep-learning1 (accessed October 26, 2022).

[40] Michelle Hauser. *Culinary Medicine Curriculum.* Chesterfield, MO: American College of Lifestyle Medicine (2019).

[41] Wolfson JA Lahne J Raj M Insolera N Lavelle F Dean M. Food agency in the United States: associations with cooking behavior and dietary intake. *Nutrients.* (2020) 12:877. 10.3390/nu12030877 32213985 PMC7146410

[42] FSHN22-9/FS445. *Best Practices for Culinary Medicine Programming.* (2022). Available online at: https://edis.ifas.ufl.edu/publication/FS445 (accessed March 26, 2023).

[43] ESHA Research. *Food Nutrition Database | Food & Ingredient Database.* (2021). Available online at: https://esha.com/nutrition-database/ (accessed November 2, 2022).

[44] Roberts JP Fisher TR Trowbridge MJ Bent C. A design thinking framework for healthcare management and innovation. *Healthcare.* (2016) 4:11–4. 10.1016/j.hjdsi.2015.12.002 27001093

[45] Stanford d.school. *An introduction to design thinking: Process guide.* Stanford, CA: Hasso Plattner Institute of Design at Stanford (2010).

[46] Dickson VV Lee C Yehle KS Abel WM Riegel B. Psychometric testing of the self-care of hypertension inventory. *J Cardiovasc Nurs.* (2017) 32:431. 10.1097/JCN.0000000000000364 27631117

[47] Silveira LCJ Rabelo-Silva ER Ávila CW Beltrami Moreira L Dickson VV Riegel B. Cross-cultural adaptation of the self-care of hypertension inventory into Brazilian Portuguese. *J Cardiovasc Nurs.* (2018) 33:289. 10.1097/JCN.0000000000000442 28731915

[48] Silveira LCJ De Maria M Dickson VV Avila CW Rabelo-Silva ER Vellone E. Validity and reliability of the self-care of hypertension inventory (SC-HI) in a Brazilian population. *Heart Lung.* (2020) 49:518–23. 10.1016/j.hrtlng.2020.02.048 32192824

[49] Ea EE Colbert A Turk M Dickson VV. Self-care among Filipinos in the United States who have hypertension. *Appl Nurs Res.* (2018) 39:71–6. 10.1016/j.apnr.2017.11.002 29422180

[50] Self-Care Measures. *Self-Care Scoring Algorithm.* (2022). Available online at: https://self-care-measures.com/self-care-scoring-algorithm/ (accessed November 22, 2022).

